# Dance/Movement Therapy and the Arts in Healthcare: The First 50 Years

**DOI:** 10.1007/s10465-016-9235-z

**Published:** 2016-10-07

**Authors:** Sherry W. Goodill

**Affiliations:** Drexel University College of Nursing and Health Professions, Philadelphia, PA USA

According to Brandman (2007), the arts in healthcare movement came into being in the 1970s in the U.S., a few years after dance/movement therapy (DMT) was defined and professionalized by the formation of the American Dance Therapy Association (ADTA) in 1966. Brandman (2007) names two organizations as forerunners to the arts in healthcare movement: Hospital Audiences, Inc., and Very Special Arts. The International Arts Medicine Association (IAMA) was founded in the mid-1980s by Dr. Richard Lippin, a poet physician and strong advocate for the creative arts therapies (CATs). The Society for Arts in Healthcare (SAH) was established in 1991, and by sometime soon after 2002 IAMA had closed its operations. The SAH was renamed the Global Alliance for Arts and Health (GAAH) and then ceased operations early in 2015, citing operational and funding challenges. However, the peer-reviewed journal established by SAH, *Arts & Health: An International Journal for Research, Policy and Practice*, has persisted, publishing steadily since its launch in 2009 and now at three issues per year. A cursory search of the 19 issues in that timeframe shows 11 article titles or abstracts that reference dance, movement, or dance/movement therapy (Taylor & Frances Online, n.d.). And a board-certified dance/movement therapist, Dr. Ilene Serlin, serves on the editorial board of that journal. The Americans for the Arts website (AFTA, n.d.) now houses some of the resources previously offered through the GAAH.

My reflections on the unfolding relationship between DMT and arts in healthcare emanate from my two primary professional roles. First, as a DMT and CATs academic with a focus on medical applications of DMT, the arts in healthcare arena has been a space in which I have been able to share my work, and a place for me to learn and network. Second, in my years as vice-president and president of the ADTA, the relationship with the arts in healthcare community proved vital to two important initiatives: (a) our integration of DMT to discussions regarding services to military service members, veterans, and their families, and (b) the team effort that is Arts Advocacy Day, an annual national-level opportunity to educate federal legislators on the CATs. It was in the two decades from the mid-1990s to the 2015 closing of the GAAH that we saw the most fruitful and dynamic interaction between DMT (as represented by the ADTA) and the arts in healthcare movement (as represented by SAH). My reflections here are limited to my experience, which does not include activity in the UK, Australia, Europe, Asia, or Africa, although there is plentiful activity, growth and scholarship in both DMT and arts in healthcare (see more in Brandman, 2007).

My own first contact with the formal arts in healthcare movement came in the mid-1990s, through Dr. Lippin, and this led to my participation in an arts in medicine conference at Shands Hospital (Goodill, 1995). One of the world’s most successful AIM programs has blossomed over the last two decades at University of Florida, and it was for this reason I invited its founder Dr. John Graham-Pole, to write the foreword to my own text on medical dance/movement therapy. At one time, there was an unmistakable and unfortunate mistrust between those in the arts in healthcare circles and CATs, but with time and mutual education, this has softened. Both groups reach for recognition and compensation in the competitive U.S. healthcare system, and there remain several areas for discussion which will require ongoing collaboration.

In the Fall 2012 edition of the ADTA Newsletter, I discussed the importance of inter-professional relationships between the ADTA and organizations representing other related fields, and ADTA has indeed invested in the relationship with our colleagues in arts in healthcare. The ADTA sent formal representation to all annual SAH conferences from 2008 to 2014. Many ADTA members have contributed to these efforts through exhibiting, presenting about DMT or related topics, serving on SAH committees and/or as representatives (of ADTA) in organizational meetings. We hosted then SAH president Dr. Gary Christenson as a special guest to the 2011 ADTA annual conference. There are several dance/movement therapists and ADTA members who have forged strong and fruitful relationships with arts in healthcare leaders and specialists, and here I name but a few of them. Notably, Dr. Ilene Serlin BC-DMT worked closely with the arts in healthcare team at the University of Florida Health Arts in Medicine team in the publication of a volume, *The Arts and Health* (Sonke-Henderson, Brandman, Serlin & Graham-Pole, 2007) part of a groundbreaking three volume series*, Whole Person Healthcare*, which Serlin edited. Among Serlin’s co-editors is Jill Sonke, who has led the University of Florida Center for Arts in Medicine (AIM) with a genuine interest in DMT and in the process of accurately describing and programming with the two approaches (i.e., the CATs with a focus on DMT and arts in healthcare with a focus on dance). Jenny Lee, BC-DMT, is actively bridging the arts in healthcare focus and DMT in the direct clinical services and education that she offers through the University of Florida AIM center. And Susie Imus, BC-DMT, has worked assiduously to describe how the two scopes of practice are at once overlapping and distinct. According to the AAH website, the range of arts in healthcare is broad, with five major foci: creating healing physical environments for health care settings, advancing community well-being through the arts, caring for professional and family caregivers through the arts, educating health care professionals through the arts, and enhancing patient care through participatory arts services (GAAH/SAH, 2011). It is in this last area, patient care, where we find the overlap and sometimes the confusion regarding the practice of CATs and arts in healthcare programming.

There has recently been concrete progress in the clarification of role boundaries and role functions. This progress is evidenced in two recent documents. The *White Paper on Arts and Health in the Military* contains an accurate definition and description of DMT and specific information on DMT credentials (NIAHM, 2013, p. 51). Second, the University of Florida website contains this well-articulated statement:

Artists in residence and arts therapists play distinct and complementary roles in a healthcare setting. An artist in residence in a healthcare setting is a practicing, professional artist in an artistic discipline such as visual art, music, dance, theatre or writing. An arts therapist is a mental health professional who utilizes an artistic discipline for a psychotherapeutic purpose. The expertise of an artist in residence is to facilitate creative process using their artistic discipline in a healthcare setting. The expertise of an arts therapist (i.e. dance therapy, art therapy, drama therapy, or music therapy) is to assess, treat and evaluate an individual using their artistic discipline of training to facilitate a psychotherapy session.

The primary distinction between the two disciplines of the Arts Therapies and the Arts in Healthcare, also known as Arts in Medicine, is the objective of engaging a patient, loved one, or clinician in the healthcare setting. An arts therapist engages a client for therapeutic goals and objectives serving as an integrated, interdisciplinary healthcare practitioner. An artist in residence engages an individual for the purpose of making art together to humanize the healthcare environment and uplift the body, mind and spirit through the experience of making art. While [artists] in residence [are] also… integrated team [members], they do not serve the healthcare team in a clinical capacity nor do they make mental health assessments or set psychotherapeutic objectives. An artist in residence has the clear and pure task of facilitating creative process; and making art. The arts therapist establishes therapeutic goals with the client; and continually engages the creative process with the aim of meeting stated goals. (UFH-AIM, n.d. FAQ)

The sorting out of scope of practice, educational requirements and certification will likely remain a salient point of attention and dialogue between the DMT community and the arts in healthcare community for the coming years. As of this writing, the development of a certification exam for arts in healthcare practitioners, now underway (AHCC, n.d.) necessitates this kind of attention and collaborative open dialogue.
